# Potential Role of TRPV4 in Stretch-Induced Ghrelin Secretion and Obesity

**DOI:** 10.1155/2022/7241275

**Published:** 2022-11-08

**Authors:** Shunsuke Hayakawa, Tatsuya Tanaka, Ryo Ogawa, Sunao Ito, Shuhei Ueno, Hiroyuki Koyama, Okubo Tomotaka, Hiroyuki Sagawa, Tomohiro Tanaka, Hiroshi Iwakura, Hiroki Takahashi, Yoichi Matsuo, Akira Mitsui, Masahiro Kimura, Satoru Takahashi, Shuji Takiguchi

**Affiliations:** ^1^Department of Gastroenterological Surgery, Nagoya City University Graduate School of Medical Sciences, Nagoya, Aichi, Japan; ^2^Department of Gastroenterology and Metabolism, Nagoya City University Graduate School of Medical Sciences, Nagoya, Japan; ^3^Department of Pharmacotherapeutics, Wakayama Medical University, Kimiidera, Wakayama, Wakayama, Japan; ^4^Department of Experimental Pathology and Tumor Biology, Nagoya City University Graduate School of Medical Sciences, Nagoya, Aichi, Japan

## Abstract

Obesity is an important health problem, which can be prevented through appetite control. Ghrelin is an appetite-stimulating hormone considered to promote obesity. Thus, we examined whether gastric stretching affects ghrelin secretion. We investigated the role of transient receptor potential vanilloid 4 (TRPV4) in gastric glands in the regulation of ghrelin secretion. TRPV4 immunostaining was performed in tissue samples from 57 patients who underwent gastrectomy. TRPV4 expression was compared between patients with (body mass index (BMI) ≥ 30) and without (BMI <30) obesity. For in vitro experiments, we used MGN3-1 cells, a ghrelin-producing cell line derived from mice. To investigate the bioactivity of TRPV4, MGN3-1 cells were treated with TRPV4 agonists and antagonists, and changes in intracellular Ca^2+^ concentration were confirmed. The concentration of ghrelin in the cell supernatant was measured using the ELISA with and without 120% stretch stimulation. TRPV4 expression was significantly higher in patients with obesity than in those without at all sites, except the fornix. Immunostaining confirmed the expression of TRPV4 in MGN3-1 cells. TRPV4 agonist administration increased intracellular Ca^2+^ concentration and ghrelin secretion in MGN3-1 cells, whereas the administration of the agonist combined with the antagonist decreased intracellular Ca^2+^ concentration and ghrelin secretion. Ghrelin secretion significantly increased in response to a 120% stretch in MGN3-1 cells. However, secretion was not increased by stretch when cells were treated with a TRPV4 antagonist. TRPV4 regulates ghrelin secretion in response to stretch in the stomach, which may affect body weight.

## 1. Introduction

Obesity is a problem prevalent worldwide; in 2015, more than 600 million people were obese, and nearly 4 million died due to obesity [1]. Obesity is a global problem that significantly impairs life prognosis. Recently, surgical treatments, such as sleeve gastrectomy, have been introduced, and treatment outcomes are improving [2, 3]. However, such a treatment is not available to everybody. Moreover, even after the treatment, there are cases that obesity does not improve sufficiently. A better understanding of appetite regulation may lead to improvements in treating morbid obesity.

Ghrelin, a gut peptide hormone, stimulates appetite [4, 5]. Ghrelin expression in the stomach is associated with the prognosis of obesity [6–9]. Moreover, the mechanism of ghrelin secretion is closely related to obesity. Dopamine, isoproterenol, oxytocin, muscarine, and prostaglandin (E2) enhance ghrelin secretion, whereas lactate, palmitate, and somatostatin inhibit ghrelin secretion [10, 11]. Chemicals, such as cinnamon, and changes in blood glucose levels also regulate ghrelin secretion, suggesting the existence of multiple regulatory mechanisms [12–14]. Ghrelin might be associated with gastrointestinal peristalsis, also known as the migrating motor complex, in humans and mice, although no conclusive results have been obtained [15, 16]. We hypothesized that stretching stimuli, such as peristalsis in the stomach, are related to ghrelin secretion, which affects body weight.

We investigated the role of the transient receptor potential vanilloid 4 (TRPV4) channel, a member of the TRP family of cation channels, as a mediator of stretching stimuli in the stomach [17]. TRPV1, which is opened by capsaicin, was the first TRPV channel to be identified; subsequently, TRPV1-6 were identified [18–20]. Notably, TRPV4 is activated by chemical stimuli, such as GSK1016790 A, heat, and mechanical stimuli [17, 21]. TRPV4 is present in the bladder epithelium and transduces the mechanical signal of the bladder wall stretching to stimulate sensory nerves [22]. TRPV4 is also present in the gastric epithelium of mice and plays a variety of roles, including ATP release in response to stretch [23, 24]. In humans, TRPV4 is expressed in gastric epithelial cell lines [25]. However, to our knowledge, the role of TRPV4 in mediating stretch stimuli has not yet been examined in the stomach. At our institution, gastric cancer surgery, esophageal cancer surgery, and sleeve gastrectomy for morbid obesity have been performed. We collected gastric tissue from patients with normal or excess body mass indexes (BMIs). These tissue samples were used to examine TRPV4 expression in the human stomach and the relationship between the TRPV4 expression and BMI. Understanding the correlation between the TRPV4 expression level and BMI may provide promising targets for new strategies to combat obesity. Furthermore, the role of TRPV4 in ghrelin secretion was investigated using MGN3-1 cells, a ghrelin-producing cell line from mice established by Iwakura et al. [26].

## 2. Materials and Methods

### 2.1. Tissue Samples from the Human Stomach

**Figure 1:**
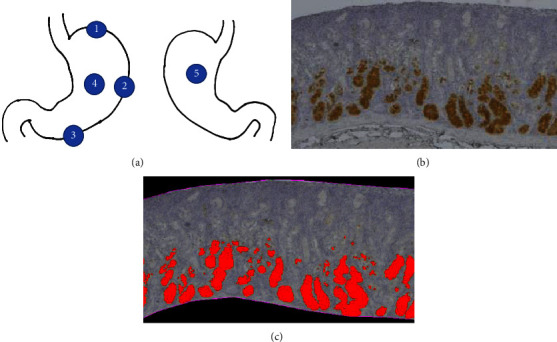
Quantification of TRPV4 expression. (a) Sites where tissue samples were taken and the quantification method for stained areas. Tissue was collected from 57 patients who underwent gastric resection. The following areas were sectioned in all layers: 1. greater curvature of the fornix; 2. greater curvature of the gastric body; 3. greater curvature of the antrum; 4. anterior wall of the gastric body; 5, posterior wall of the gastric body. (b) TRPV4 immunostaining in gastric tissue. (c) The images were captured using a microscope with BZ-X710 (Keyence, Osaka, Japan), and staining was quantified with hybrid cell count BZ-H4C.

We collected tissue samples from 57 patients who underwent esophageal or gastric surgery at Nagoya City University Hospital (Aichi, Japan) between September 2019 and November 2020. All patients who consented to the tissue collection during the same period were included in the study. Overall, 14 patients underwent laparoscopic sleeve gastrectomy (all cases were of morbid obesity), 3 patients underwent total gastrectomy (3 cases of gastric cancer), 16 patients underwent distal gastrectomy (15 cases of gastric cancer and 1 case of gastric ulcer), 3 patients underwent proximal gastrectomy (2 cases of gastric cancer and 1 case of gastric ulcer), and 21 patients underwent subtotal esophagectomy (20 cases of esophageal cancer and 1 case of esophagostenosis). We collected gastric tissue samples from five sites for immunohistochemistry staining: the greater curvature of the antrum (5 cm from the pylorus), the greater curvature of the gastric body, the fornix, the anterior wall of the gastric body, and the posterior wall of the gastric body (see Figure 1(a)). Because total gastrectomies were rare, we only collected tissue samples from areas that could be removed according to the surgical technique and were not involved in cancer localization. After fixation in buffered 10% formalin, we collected tissue samples from the same five sites as above for immunostaining. The samples were paraffinized, sectioned, and mounted on slides.

The study was approved by the Ethics Review Committee of Nagoya City University (approval no. 60-19-0038) and written informed consent was obtained from all patients. All study procedures were performed in accordance with the ethical standards laid down in the 1964 Declaration of Helsinki and its later amendments.

### 2.2. Analysis of Immunohistochemical Staining of Human Gastric Tissue

Dewaxed paraffin sections were placed in a microwave (10 min, 600 watts) to recover antigens before staining. The tissue samples were probed with rabbit polyclonal anti-TRPV4 antibodies (ab94868, Abcam, Cambridge, UK; 1 : 500) overnight at 4°C. On the next day, peroxidase-conjugated streptavidin was used with 3,3-diaminobenzidine tetrahydrochloride (DAB; Biocare Medical, Concord, CA, USA) for antibody detection. Hematoxylin was used for nuclear counterstaining. Renal tissue sections were used as positive controls for TRPV4-immunostaining, whereas negative controls were stained without the primary antibody. Images of the stained slides were captured using a BZ-X710 fluorescent microscope (Keyence Corporation, Osaka, Japan). We quantified the stained areas using hybrid cell count software (BZ-H4C, Keyence Corporation, Osaka, Japan) (see Figures 1(b) and 1(c)).

### 2.3. Comparison between Obese and Nonobese Cases

Obesity was defined according to the World Health Organization criteria, with a BMI of 30 as the cutoff. 57 study participants were classified into two groups: 15 cases in the obese group with BMI ≥30 (O group) and 42 cases in the nonobese group with BMI <30 (N group). We compared the area of TRPV4 immunostaining in the O and N groups at each stomach site. The median area of TRPV4 staining in the greater curvature of the gastric body in the N group was defined as 1.0, and the ratios were compared.

### 2.4. Immunohistochemical Staining for *H. pylori* in Gastric Tissue

Dewaxed paraffin sections were placed in a microwave (10 min, 600 watts) to recover antigens before staining. The tissue samples were probed with rabbit anti-*H. Pylori* antibodies (B0471, DAKO, Glostrup, Denmark; 1 : 200) using Dako Autostainer Link 48(DAKO). Peroxidase-conjugated streptavidin was used with 3,3-diaminobenzidine tetrahydrochloride for antibody detection, and hematoxylin was used for nuclear counterstaining. Stained slides were analyzed for the presence of the bacteria by an experienced pathologist.

### 2.5. Cell Culture

The ghrelin-producing MGN3-1 cell line was isolated from a gastric tumor of ghrelin promoter simian virus 40 T-antigen transgenic mice (kindly provided by Dr. Hiroshi Iwakura, Wakayama Medical University, Wakayama, Japan). MGN3-1 cells were cultured in DMEM supplemented with 10% fetal bovine serum (FBS), 100 U/ml penicillin, and 100 *μ*g/ml streptomycin at 37°C in 10% CO_2_, as described previously [26].

### 2.6. Reverse Transcription (RT)-PCR Analysis in MGN3-1 Cells

MGN3-1 cells were seeded at 0.6 × 10^7^ in six-well plates containing 5% bovine serum albumin. After 24 h, cell pellets were harvested, total RNA was extracted from cell pellets using an RNeasy Plus Mini Kit (Qiagen, TX, USA) according to the manufacturer's protocols and quantified using NanoDrop^TM^1000 (Thermo Fisher Scientific, Inc.). RT was performed using SuperScript III First-Strand Synthesis SuperMix for qRT-PCR (Invitrogen, CA, USA). We performed RT-PCR with TaqMan probes (TRPV4 : Mm00499025_m1; GAPDH : Mm99999915_g1 (Applied Biosystems, CA, USA)) and TaqMan Universal Master Mix (Applied Biosystems) using CFX Connect Real-Time System (Bio-Rad Laboratories, Inc.). The PCR program consisted of initial denaturation at 95°C for 20 s followed by 60 cycles of 95°C for 1 s and 60°C for 20 s.

### 2.7. Immunocytochemical Analysis of TRPV4 in MGN3-1 Cells

MGN3-1 cells were seeded at 1 × 10^5^ cells/chamber in an 8-chamber glass slide and cultured overnight. The cells were then washed with PBS and fixed with 4% paraformaldehyde for 20 min at room temperature. The cells were washed and permeabilized with 0.1% Triton-X for 3 min and incubated with blocking buffer (3% BSA, FUJIFILM Wako Pure Chemical Corporation, Tokyo, Japan) for 1 h at room temperature. The cells were then probed with a rabbit polyclonal anti-TRPV4 antibody (OST00265 G, Osenses Pty Ltd, Keswick, Australia) overnight at 4°C. Subsequently, the cells were washed and incubated with ALexa Fluor® 488 goat anti-rabbit IgG (ab150077, Abcam, Cambridge, UK) for 1 h at room temperature. Primary and secondary antibodies were used at 1 : 500 dilutions with 3% BSA. The nuclei were visualized with 4′,6-diamidino-2-phenylindole staining at room temperature for 10 min. Images of the stained slides were captured using a BZ-X710 fluorescent microscope (Keyence Corporation, Osaka, Japan).

### 2.8. Intracellular Calcium Measurement

MGN3-1 cells were seeded at 5 × 10^4^ cells/well and cultured overnight in 96-well plates. Intracellular Ca^2+^ levels ([Ca^2+^]_i_) were measured using Calcium Kit II Fluo4 (Dojindo, Kumamoto, Japan). Calcium imaging movies were recorded and analyzed using a BZ-X710 fluorescent microscope (Keyence Corporation, Osaka, Japan) at room temperature (20°C). The mean value of 20 randomly selected cells was calculated.

### 2.9. Stimulation of MGN3-1 Cells with TRPV4 Agonists and Antagonists and Measurement of Ghrelin Secretion

MGN3-1 cells were seeded at 7.5 × 10^5^ cells/well and cultured overnight in 12-well plates. After washing with PBS, the cells were incubated at 37°C in DMEM supplemented with 0.5% BSA and simultaneously stimulated with GSK1016790 A (Sigma, St. Louis, MO, USA, TRPV4 agonist, 3 *μ*M) alone or with GSK1016790 A and HC067047 (Sigma, St. Louis, USA, specific TRPV4 antagonist, 20 *μ*M) for 4 h.

The culture media were collected, centrifuged at 400 × g for 5 min to discard particulates, and stored at −80°C until further use. There are two types of ghrelin, acyl ghrelin and des-acyl ghrelin. The effects of the two types of ghrelin are different. Acyl ghrelin is active ghrelin [27]. We measured the secretion of acyl and des-acyl ghrelin using an ELISA kit (SCETI, Tokyo, Japan).

### 2.10. Mechanical Stimuli to MGN3-1 Cells and Measurement of Ghrelin Secretion

For mechanical stimuli, stretching was applied with an STB-10 stretch machine (STREX, Tokyo, Japan). Stretch chambers were coated with type I collagen (Nitta-gelatin, Osaka, Japan, Cellmatrix Type I–C). MGN3-1 cells were seeded at 7.5 × 10^5^ cells/well and cultured overnight in the chambers. After washing with PBS, the cells were subjected to stretching; the stretching protocol consisted of two courses of 9 × 1.2 stretches for 3 min in both courses with a two-hour rest period between the two courses. One chamber was subjected to the same stretching protocol and treatment with HC067047 (Sigma; 20 *μ*M). One chamber with a vehicle without any stretching acted as a control. Secreted acyl ghrelin and des-acyl ghrelin were measured by the ELISA (SCETI, Tokyo, Japan).

### 2.11. Data Analysis

Data are reported as medians with 25^th^ and 75^th^ percentiles for gastric tissue sample measurements or means ± standard error of the mean for in vitro experiments. The Mann–Whitney *U* test was used to compare data between the two patient groups because clinical data were not normally distributed. Fisher's exact test was used when the ratio (frequency) test included parametric values. Comparisons between multiple groups were performed using one-way analysis of variance with Bonferroni's post hoc test to perform subsequent comparisons of individual groups for vitro data. A *p* value of <0.05 was considered statistically significant. Statistical analyses were performed using EZR [28].

## 3. Results

### 3.1. Comparison of TRPV4 Expression in Gastric Tissue from Obese and Nonobese Cases

**Figure 2:**
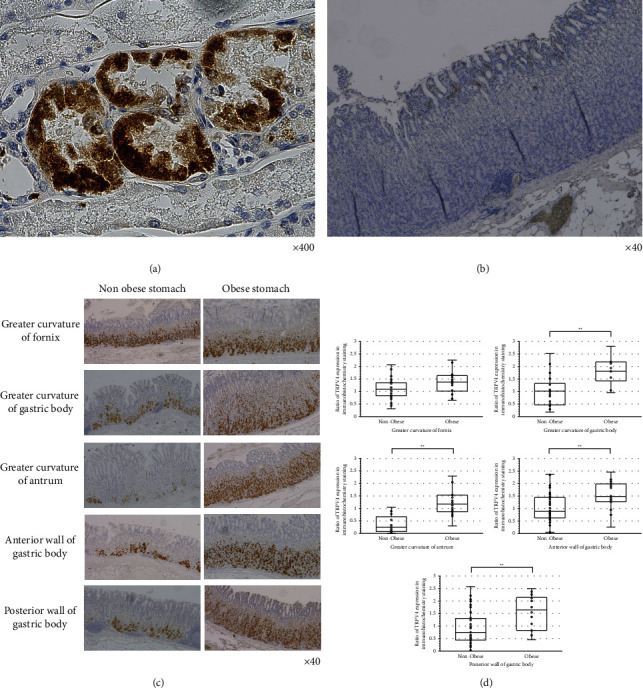
Comparison of stained areas between nonobese and obese cases. (a) Renal tissues were used as a positive control for TRPV4-immunostaining. (b) For the negative control, tissues were subjected to immunostaining without the primary antibody. (c) Immunostaining of the nonobese and obese stomach. (d) Comparison of stained areas in tissue samples collected from patients with and without obesity. Lines within the boxes represent median values; upper and lower lines of the boxes represent 25th and 75th percentiles, respectively; upper and lower bars outside the boxes represent the maximum and minimum, respectively ( ^*∗∗*^*p* < 0.01).

The results of TRPV4-immunostaining for positive and negative controls are presented in Figure 2(a) and 2(b), respectively.

TRPV4 expression in the stomach is shown in Table 1 and Figures 2(c) and 2(d). In the greater curvature of the fornix, there was no significant difference in the expression of TRPV4 between the N and O groups (*p*=0.0811). The expression of TRPV4 was significantly higher in the O group than that in the N group in the greater curvature of the gastric body (*p*=0.0016), greater curvature of the antrum (*p*=0.0003), anterior wall of the gastric body (*p*=0.0060), and posterior wall of the gastric body (*p*=0.0066).

### 3.2. *Hericobacter pylori* Infection in Gastric Tissue

Overall, 3 out of 15 patients with obesity and 3 out of 42 patients without obesity were infected by *H. pylori*, and the difference between the two groups was not significant (*p*=0.18).

### 3.3. TRPV4 Expression in MGN3-1 Cells

RT-PCR test results confirmed the expression of TRPV4 in MGN3-1 cells (see Supplemental Figure 1).

**Figure 3:**
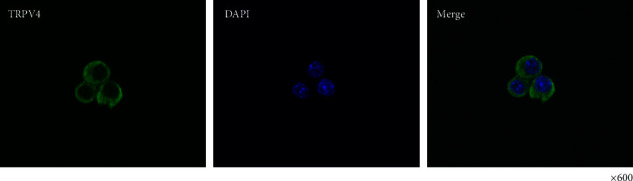
Fluorescence immunostaining of TRPV4 in MGN3-1 cells. TRPV4 protein expression was observed in MGN3-1 cells. Magnification: ×600. TRPV4-mediated change in [Ca^2+^]_i_ in MGN3-1 cells.

TRPV4 localization in MGN3-1 cells was confirmed by immunochemical staining (see Figure 3).

### 3.4. TRPV4-mediated change in [Ca^2+^]i in MGN3-1 cells

**Figure 4:**
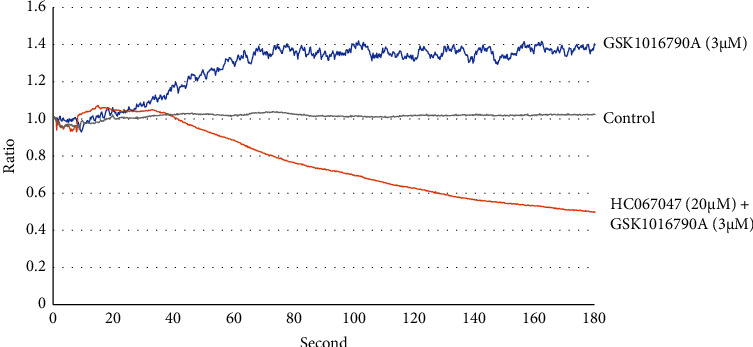
TRPV4-mediated changes in cytosolic Ca^2+^ in MGN3-1 cells. The effects of GSK1016790 A and HC 067047 on [Ca^2+^]_i_ in MGN3-1 cells. Compared to the control level at 180 s, [Ca2+]_i_ increased in MGN3-1 cells treated with GSK1016790 A (*n* = 20, *p* < 0.01) but decreased in cells treated with GSK1016790 A and HC067047 (*n* = 20, *p* < 0.01) (GSK1016790 A, TRPV4 agonist; HC067047, TRPV4 antagonist).

Treatment with the TRPV4 agonist GSK1016790 A (3 *μ*M) increased [Ca^2+^]_i_ in MGN3-1 cells. At 180 s, [Ca2+]_i_ was higher in MGN3-1 cells treated with GSK1016790 A than in control cells (*n* = 20, *p* < 0.01), whereas the [Ca2+]_i_ level decreased following treatment with GSK1016790 A and HC067047 (*n* = 20, *p* < 0.01, see Figure 4). These results indicate that TRPV4 regulates [Ca^2+^]_i_ in MGN3-1 cells.

### 3.5. TRPV4 Regulation of Ghrelin in MGN3-1 Cells

**Figure 5:**
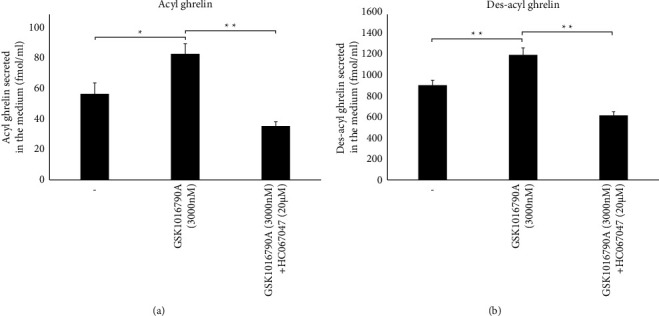
Regulation of ghrelin secretion by the TRPV4 agonist and antagonist in MGN3-1 cells. (a) The amount of secreted acyl ghrelin after four-hour treatment of MGN3-1 cells with GSK1016790 A (3 *μ*M) or HC067047 (20 *μ*M) + GSK1016790 A (3 *μ*M). (b) The amount of secreted des-acyl ghrelin after four-hour treatment of MGN3-1 cells with GSK1016790 A (3 *μ*M) or HC067047 (20 *μ*M) + GSK1016790 A (3 *μ*M) ( ^*∗*^*p* < 0.05;  ^*∗∗*^*p* < 0.01; *n* = 6; error bars: standard error of the mean).

The effects of TRPV4 agonist and antagonist treatment on acyl ghrelin and des-acyl ghrelin secretion were measured. Acyl ghrelin secretion increased in response to treatment with the TRPV4 agonist (GSK106790 A, 3 *μ*M) but decreased after cotreatment with the TRPV4 antagonist (HC067047, 20 *μ*M) and TRPV4 agonist (GSK106790 A, 3 *μ*M) (see Figure 5(a)) (control: 57 ± 7 fmol/ml, *n* = 6; GSK106790 A: 83 ± 7 fmol/ml, *n* = 6, *p* < 0.01 vs. control and *p* < 0.05 vs. GSK106790 A + HC067147; GSK106790 A + HC067147 : 36 ± 3 fmol/ml, *n* = 6). Des-acyl ghrelin secretion increased in response to 3 *μ*M of GSK106790 A but decreased in response to cotreatment with 3 *μ*M of GSK106790 A and 20 *μ*M of HC067047 (see Figure 5(b)) (control: 915 ± 44 fmol/ml, *n* = 6; GSK106790 A: 1208 ± 63 fmol/ml, *n* = 6, *p* < 0.01 vs. control and *p* < 0.01 vs. GSK106790 A + HC067147; GSK106790 A + HC067147 : 624 ± 32 fmol/ml, *n* = 6).

### 3.6. Regulation of Ghrelin Secretion by Mechanical Stimuli in MGN3-1 Cells

**Figure 6:**
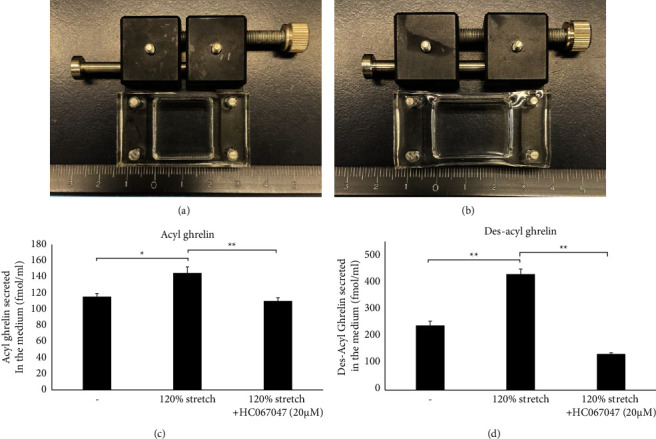
Regulation of ghrelin secretion by mechanical stimuli in MGN3-1 cells (ELISA). Cells were subjected to a 120% stretch nine times for 3 min twice. This stretch protocol was repeated 2 hours later. After 4 hours, acyl ghrelin and des-acyl ghrelin were measured by the ELISA. (a) Unstretched MGN3-1 cells. (b) 120% stretch of MGN3-1 cells. (c) The amount of acyl ghrelin in the supernatant obtained after 120% stretch and treatment of MGN3-1 cells with HC067047 (20 *μ*M) + 120% stretch. (d) The amount of des-acyl ghrelin in the supernatant obtained after 120% stretch and treatment of MGN3-1 cells with HC067047 (20 *μ*M) + 120% stretch. (^*∗*^*p* < 0.05;  ^*∗∗*^*p* < 0.001; *n* = 5; error bar: standard error of the mean).

Next, we examined the effect of a 120% stretch on ghrelin secretion. Acyl ghrelin secretion increased significantly in response to a 120% stretch (see Figures 6(a) and 6(b)) but did not change when cells were subjected to 120% stretch stimulation and treated with 20 *μ*M HC067047 (see Figure 6(c)) (control: 116 ± 4 fmol/ml, *n* = 5; 120% stretch: 145 ± 8 fmol/ml, *n* = 5, *p* < 0.05 vs. control and *p* < 0.01 vs. 120% stretch + HC067147; 120% stretch stimulation + HC067147 : 110 ± 4 fmol/ml). Des-acyl ghrelin secretion increased in response to a 120% stretch but decreased in response to a 120% stretch and 20 *μ*M HC067047 (see Figure 6(d)) (control: 239 ± 16 fmol/ml, *n* = 5; 120% stretch stimulation: 429 ± 19 fmol/ml, *n* = 5, *p* < 0.01 vs. control and *p* < 0.01 vs. 120% stretch + HC067147; 120% stretch + HC067147 : 134 ± 4 fmol/ml, *n* = 5). Although stretch increased ghrelin secretion, the addition of HC067147 did not increase ghrelin secretion more than stretch alone. These findings suggest that stretch increases ghrelin secretion in MGN3-1 cells via TRPV4 channels.

## 4. Discussion

The relationship between gastric stretch and appetite is poorly understood. We focused on the TRPV4 channel, which is activated by stretching [29–31]. Through immunostaining of human gastrectomy tissue samples, we confirmed that TRPV4 channels are expressed in human gastric glands. To the best of our knowledge, this is the first report regarding the presence of TRPV4 at the protein level in the human stomach. Furthermore, we showed for the first time that TRPV4 is widely expressed in the stomach of patients with obesity. These results indicate a relationship between TRPV4 expression in the stomach and the development of obesity. We previously confirmed that TRPV4 is expressed in the gastric glands of mice and humans (this result in mice was not shown). The prevalence of *H. pylori*, a possible factor related to TRPV4 expression, did not differ between patients with and without obesity in the present study. We also demonstrated that TRPV4 is expressed in MGN3-1 cells, a ghrelin-producing cell line derived from mice, and TRPV4 regulates intracellular Ca^2+^ concentration. We hypothesized that stretching affects ghrelin production via TRPV4 and controls appetite. TRPV4 agonists and stretch increased ghrelin secretion in MGN3-1 cells, and the addition of TRPV4 antagonists suppressed ghrelin secretion. Thus, TRPV4 in the stomach may influence body weight by regulating ghrelin secretion induced by stretch.

Cells are exposed to various physical stimuli. For example, in recent years, the role of physical stimuli in the heart has been investigated [32, 33]. Moreover, the stomach is exposed to physical stimuli during the digestion of food and peristalsis. However, the role of physical stimuli in the stomach is poorly understood [23]. Ghrelin secretion exhibits diurnal variation in humans, and secretion is elevated before meals [34, 35]. Moreover, peristaltic contractions, also called the migrating motor complex, occur in the stomach between meals [36]. Recent studies have suggested that gastrointestinal hormones, such as ghrelin, influence gastrointestinal peristalsis [15, 16, 37, 38]. However, there is concern regarding relationships between gastrointestinal hormones and gastric extension during food ingestion. Because gastric stretching during feeding involves receptivity relaxation, we consider the effect of temporary gastric stretching during oral intake to be small. Conversely, peristalsis between meals involves the migrating motor complex, which provides repeated stretching stimuli to organs [36]. Moreover, peristaltic activity between meals might provide appropriate physical stimuli to activate TRPV4 repeatedly. We assume that stretching caused by peristalsis between meals stimulates ghrelin secretion via TRPV4. This mechanism needs to be verified in the future.

In this study, we compared gastric TRPV4 expression in obese (BMI ≥30) and nonobese (BMI <30) cases. The expression of TRPV4 in the greater curvature of the fornix was similar in both the groups. In contrast, compared with the expression in patients without obesity, TRPV4 expression was higher in patients with obesity in the greater curvature of the gastric body, the greater curvature of the antrum, the anterior wall of the gastric body, and the posterior wall of the gastric body. These results suggest that TRPV4 is widely expressed in the stomach of patients with obesity. Miyazaki et al. found that the number of ghrelin granules in gastric tissue is correlated with the BMI. This finding is consistent with that of our study of increased ghrelin production in patients with obesity with widespread TRPV4 expression [7].

MGN3-1 cells are an ideal in vitro tool to study ghrelin secretion [26]. TRPV4 is a cation channel that passes Ca^2+^ and changes intracellular Ca^2+^ concentrations [39]. In MGN3-1 cells, increased intracellular Ca^2+^ affects the Gq pathway and increases ghrelin secretion [11]. We treated MGN3-1 cells with GSK1016790 A, a TRPV4 agonist, and HC067147, a TRPV4antagonist, and measured changes in intracellular Ca^2+^ concentrations [40]. Intracellular Ca^2+^ concentrations were elevated in response to GSK1016790 A and decreased in response to GSK1016790 A and HC067147 treatment. These results demonstrated that TRPV4 regulates Ca^2+^ concentration in MGN3-1 cells. Oxytocin and muscarinic drugs also affect the intracellular Ca^2+^ concentration in MGN3-1 cells. These drugs cause a transient increase in Ca^2+^ concentration within 1 min after administration, and intracellular Ca^2+^ returns to the initial concentration after 3 min [11]. In contrast, Ca^2+^ concentration increased slowly and remained high for some time after TRPV4 stimulation. Other studies have shown similar sustained changes in Ca^2+^ levels in response to TRPV4 [41, 42]. Considering that Ca^2+^ concentrations were decreased by HC067147 administration, TRPV4 regulates Ca^2+^ concentrations in MGN3-1 cells.

We examined whether ghrelin secretion changed in MGN3-1 cells in response to peristaltic-like stretches. The results showed that multiple 120% stretch stimulations increased ghrelin secretion, and the increase was inhibited by HC067147. Both acyl ghrelin and des-acyl ghrelin similarly increased. These results suggest that stretching regulates ghrelin secretion via TRPV4. Considering that TRPV4 expression increased in the stomach of patients with obesity, we predict that this mechanism may affect body weight.

The present study suggests that appropriate stretch stimulation of the stomach affects ghrelin secretion via TRPV4. These results may lead to the development of drugs that regulate appetite and decrease obesity. Immunostaining of the human stomach shows that TRPV4 is expressed in the entire gastric gland. Further study of the role of TRPV4 in the stomach may clarify some of the mechanisms of appetite regulation and digestive function.

Nowadays, the stomach is preserved as much as possible during gastrectomy, considering weight loss [43, 44]. In the gastric tissue samples used in this study, there were only two cases of total gastrectomy. The background of patients differed according to the site of the stomach because patients with different diseases were included in the study. The stomach tissue samples used as controls were resected due to other diseases, such as cancer. We cannot exclude the possibility that the presence of cancer may have affected the results. This is a limitation of the present study. However, this limitation was difficult to avoid as it is not possible to resect the stomach of a healthy person. In this study, we used mouse-derived MGN3-1 cells. Mice have different feeding intervals from humans. Furthermore, mice do not have motilin, suggesting that the role of ghrelin may differ between humans and mice [45]. Regarding the mechanism responsible for body weight changes in TRPV4 knockout mice, there are reports that high-fat diet consumption increases body weight, whereas other reports indicate that TRPV4 in organs other than the stomach may also be affected. It is currently controversial whether body weight would change if TRPV4 levels were inhibited systemically [46, 47]. In 2019, a study on heart failure with oral TRPV4 antagonists on humans was conducted; however, there were no reports of weight gain or loss [48]. To the best of our knowledge, there are no studies regarding the oral administration of TRPV4 agonists or antagonists in mice. The development of orally administrable drugs is needed in the future.

In the present study, GSK106790 A and HC067147 were used at concentrations that were higher than those used in other reports. This might be a limitation of the present study. In cell extension experiments, a small number of cells were detached from the chamber during extension stimulation. However, because ghrelin secretion increased only upon extension stimulation of cells that were not detached, the overall results of the experiment were unlikely to be affected. Although this is another limitation, this event cannot be prevented in extension experiments.

## 5. Conclusions

The findings of the study suggest that TRPV4 in the stomach regulates ghrelin secretion in response to stretch and affects body weight.

## Figures and Tables

**Table 1:** TRPV4 immunostaining in the stomach of patients with and without obesity.

Site of stomach	Nonobese cases	Obese cases	*p* value
Greater curvature of the fornix	1.10 (0.85, 1.33)	1.38 (1.05, 1.61)	0.0811
Greater curvature of the gastric body	1.00 (0.48, 1.31)	1.80 (1.55, 2.16)	0.0016 ^*∗*^
Greater curvature of the antrum	0.24 (0.08, 0.55)	1.17 (0.89, 1.48)	0.0003 ^*∗*^
Anterior wall of the gastric body	0.88 (0.64, 1.40)	1.49 (1.29, 1.94)	0.0060 ^*∗*^
Posterior wall of the gastric body	0.74 (0.46, 1.28)	1.65 (0.90, 2.09)	0.0066 ^*∗*^

*Note.* Data are expressed as a median (25th percentile and 75th percentile),  ^*∗*^*p* < 0.01.

## Data Availability

The data that support the findings of this study are available on request from the corresponding author. The data are not publicly available due to privacy or ethical restrictions.

## Supplementary Materials

Supplementary Materials Supplemental Figure 1. Amplification curves for RT-PCR using MGN3-1 cells (blue line: GAPDH, red line: TRPV4; four wells each).
