# Supplementary figures and images for: Potential Role of TRPV4 in Stretch-Induced Ghrelin Secretion and Obesity
Source: Int J Endocrinol. 2022 Nov 8;2022:7241275. doi: 10.1155/2022/7241275 (PMC9666045; doi:10.1155/2022/7241275)

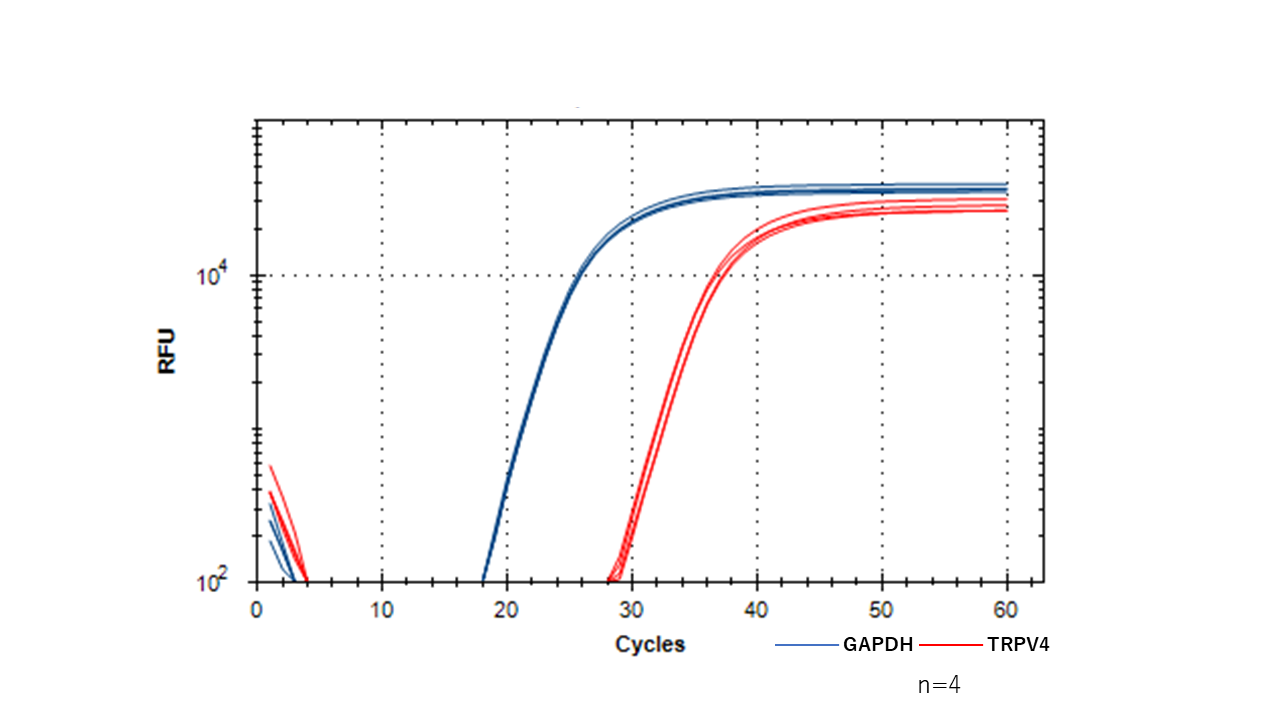

Supplement: Supplementary Materials — Supplemental Figure 1. Amplification curves for RT-PCR using MGN3-1 cells (blue line: GAPDH, red line: TRPV4; four wells each). [file 7241275.f1.zip › Supplemental Figure1 (1).jpeg]
